# Bradykinin promotes migration and invasion of human immortalized trophoblasts

**DOI:** 10.1186/1477-7827-9-97

**Published:** 2011-07-05

**Authors:** Rafaela Erices, Jenny Corthorn, Francisco Lisboa, Gloria Valdés

**Affiliations:** 1 Centro de Investigaciones Médicas, Escuela de Medicina Pontificia Universidad Católica, Santiago, Chile; 2 Departamento de Nefrología, Escuela de Medicina Pontificia Universidad Católica, Santiago, Chile

## Abstract

Having demonstrated that the bradykinin B2 receptor (B2R) is expressed in cells that participate in trophoblast invasion in humans and guinea-pigs, we investigated the role of bradykinin (BK) on cell migration and invasion in the HTR-8/SVneo trophoblast cell line using wound healing and invasion assays. First, we documented that HTR-8/SVneo cells expressed kallikrein, B2R, B1R, MMP-2 and MMP-9 using immunocytochemistry. Incubation with BK (10.0 microMol/L) for 18 hours increased the migration index 3-fold in comparison to controls or to cells preincubated with the B2R antagonist HOE-140. BK (10.0 microMol/L) incubation yielded a similar number of proliferating and viable cells as controls, therefore the enhanced closure of the wound cannot be attributed to proliferating cells. Incubation with BK (10.0 microMol/L) for 18 hours increased the invasion index 2-fold in comparison to controls or to cells preincubated with the antagonist of the B2R. Neither the B1R ligand Lys-des-Arg9 BK, nor its antagonist Lys-(des-Arg9-Leu8), modified migration and invasion. Further support for the stimulatory effect of B2R activation on migration and invasion is provided by the 3-fold increase in the number of filopodia per cell versus controls or cells preincubated with the B2R antagonist. Bradykinin had no effect on the cellular protein content of the B2R, nor the MMP-9 and MMP-2 gelatinase activity in the culture media varied after incubation with BK. This study adds bradykinin-acting on the B2R-to the stimuli of trophoblast migration and invasion, an effect that should be integrated to other modifications of the kallikrein-kinin system in normal and pathological pregnancies.

## Background

In humans, the invasion of maternal decidua and uterine spiral arteries by the extravillous trophoblast (EVT) is essential for the establishment of a normal placenta and adequate blood flow to the fetus. On the maternal side, EVT invasion is initiated when cytotrophoblasts anchor the budding placental villi to the uterine wall; in a second stage, EVTs detach and migrate across the endometrium, transform the arteries, and finally settle in their lumen [1-3]. Simultaneously, on the fetal side, villous cytotrophoblasts establish a richly branching tree that provides the extensive surface in which fetal and maternal blood exchange nutrients and waste products.

Dysregulation of trophoblast invasion is associated with various pathologies, such as intrauterine growth retardation, preterm birth, placenta accreta and the preeclampsia syndrome, its foremost clinical manifestation [4-7]; all these complications increase maternal and fetal morbidity and mortality. Over the years proteolytic, adhesive, growth promoting, inflammatory and angiogenic molecules that modulate trophoblast migration and invasion have been identified, and localized in trophoblasts, maternal epithelial and stromal cells, uterine NK cells and macrophages [8-13]. The list of factors controlling trophoblast invasion in normal placentation is continuously expanding, but despite intensive research, our understanding of normal and pathological processes remains limited.

It has been postulated that nitric oxide (NO) regulates trophoblast invasion by priming the maternal blood vessels [1,14]. We have hypothesized that NO integrates a network of vasodilator systems including the kallikrein-kinin system (KKS), which includes serine proteases, and tissue and plasma kallikrein (Kal), that generate kallidin and bradykinin from low and high molecular kininogen, respectively [15]. The upregulation of Kal and endothelial nitric oxide synthase (eNOS) in placenta accreta, a condition of exaggerated trophoblast invasion, suggested that vasodilators facilitate trophoblast migration [16]. However, the tissue KKS, initially considered a vasoactive system, is now known to have pleiotropic effects which deserve to be studied in pregnancy.

Bradykinin (BK)-related peptides activate G-protein coupled receptors, the bradykinin type 1 and type 2 receptors (B1R and B2R) [17,18]. B1Rs are inducible, and their natural agonist lacks the C-terminal Arg residue of BK; they cause chronic inflammation, pain, hypotension and proliferation of tumoral cells. They are exceptionally constitutive in the central nervous system, and information on their actions is derived from pharmacological studies. B2Rs are constitutive, require the full peptide chain and activate endothelial cells leading to vasodilatation, increased vascular permeability, production of NO, and mobilization of arachidonic acid. They are localized in endothelial cells, smooth muscle, fibroblasts, mesangial cells, neurons, astrocytes, and polynuclear neutrophils. In reproductive tissues the B2R has been documented in decidua, placental and extravillous trophoblasts, and in the fetal endothelium in humans, rats and guinea-pigs [19-23].

Bradykinin stimulates cell migration, a critical process in placentation, embryogenesis, wound healing, immune response, tissue development, vascular disease and cancer [24-27]. BK increases migration of endothelial cells [28], endothelial progenitor cells [29], neutrophils [30,31], lymphocytes [32], fibroblasts [33], dendritic cells [34], microglia [35], and cancer cells [36-38]. Interestingly, BK has been found to induce the formation of peripheral actin microspikes, filopodia and lamellipodia in fibroblasts and endothelial progenitor cells, indicating its relevance in determining an invasive phenotype [33]. Whether the effects are mediated by the B1R or the B2R depends on the different cell types.

In addition, activation of the BK receptors stimulates metalloproteinases, key molecules in trophoblast invasion. In rat, astroglial cell line activation of the B2R modulates MMP-9 gene expression and cell migration by phosphorylating and translocating the protein kinase-delta-dependent extracellular kinase1/2, which in turn activates its downstream factor Elk-1 [39]. On the other hand, B1R stimulation induces release of MMP-2 and MMP-9 via an ERK-dependent pathway in estrogen-sensitive and-insensitive breast cancer cells [37].

The aim of the present study was to evaluate the effect of bradykinin on the migratory and invasive capacity of HTR-8/SVneo cells, an immortalized line of first trimester extravillous trophoblast, which constitutes a valid model for extravillous trophoblasts [40-43]. First, we confirmed the main components of the KKS in these cells. We identified the B2R as the receptor involved in the BK-stimulated migratory and invasive capacity of these cells. Lastly, we demonstrated that BK induced filopodias, a characteristic conformation of the cytoskeleton in migrating cells.

## Methods

### HTR-8/SVneo cell culture

HTR-8/SVneo trophoblast cells were kindly donated by C.H. Graham, Queen's University, Kingston, Canada. The cells were obtained from first trimester human placenta (8-10 wk gestation) and immortalized by transfection with a cDNA construct that encodes the simian virus 40 large T antigen. Though nontumorigenic and nonmetastatic, the cells are highly invasive in vitro and exhibit phenotypic properties of extravillous cytotrophoblasts, including the expression of cytokeratins 7, 8, and 18, placental alkaline phosphatase, urokinase-type PAR, human leukocyte antigen (HLA) framework antigen W6/32, IGF-II mRNA and protein, and the integrin profile characteristic of invasive cytotrophoblasts [42]. When plated on Matrigel these cells express HLA-G [44].

Cells were cultured as described [40,41]. Briefly, cells were grown in RPMI 1640 (Sigma-Aldrich) supplemented with 10% FBS (Gibco) and 50 μg/ml gentamicin (Gibco) in a humidified atmosphere of 95% air and 5% CO2 at 37°C. Cells at passage 6-25 were used to perform all the experiments.

### Immunocytochemistry

Immunocytochemistry was performed for kininogen, Kal, B1R, B2R, MMP-2, MMP-9, cytokeratin and Ki67. The dilution and origin of the antibodies were: anti-kininogen (1:500, Boehringer); anti-Kal (1:2,000, produced in our laboratory [45]); anti-B1R (1:1,000, Santa Cruz Biotechnology), and anti-B2R mouse monoclonal (1:4,000, BD Transduction Laboratories); anti-MMP-2 and anti-MMP-9 (1:20 and 1:200 respectively, Calbiochem mouse mAb, USA); anti-pancytokeratin mouse monoclonal, (1:100, Sigma-Aldrich) and anti-Ki67 mouse monoclonal (1:50, BioGenex).

Cells were seeded at a density of 80,000 cells/well on cover slides in 24-well plates (Nucleon Surface, Nunc), in RPMI 1640, supplemented with 1% FBS, 50 μg/ml gentamicin in a humidified atmosphere of 95% air and 5% CO_2 _at 37°C. After 18 hours, cells were rinsed 3 times with cold PBS and fixed with methanol at -20°C for 20 minutes, equilibrated 3 times with 50 mMol/L TRIS-HCl PBS buffer pH 7.8 and incubated with 10% H_2_O_2 _for 5 minutes to block endogenous peroxidases. Cells were incubated with protein block (Cas-Block, Zymed Laboratories) for 30 minutes in a humidified chamber, and incubated with the respective antibodies at the aforementioned concentrations for 18 hours at 4°C. Cells were immunostained with the biotin-streptavidin peroxidase system (Dako LSAB+System-HRP), incubated with 0.1% of 3-3'-diaminobenzidine (Sigma-Aldrich) in the presence of 0.05% H_2_O_2 _for 15 minutes, stained with Harris Haematoxylin (Sigma-Aldrich) and finally mounted with glycerin-gelatin (Kaisers, Merck).

To determine whether kininogen-the substrate from which kallikrein generates bradykinin-is an inherent or a trapped molecule, cells were incubated with or without serum for 24-hours. The specificity of the staining was determined by incubating sections in the absence of the first antibody, or in the presence of rabbit IgG fraction (1:50 to 1:1000) and mouse IgG serum (1:50 to 1:1,000, both from Dako Cytomation), depending on the species in which the antibodies were generated.

### Western blot analysis

Total proteins from HTR-8/SVneo cells were extracted using 20 mM Tris-HCl buffer containing 10 mM EDTA, 2 mM phenylmethylsulfonylfluoride, 5 μM leupeptin, 50 μg/ml soybean trypsin inhibitor and 0.02% NaN3 at pH 7.4. Lysed cells were left for 20 minutes on ice, centrifuged at 14,000 rpm for 20 minutes at 4°C and finally stored at -70°C. Protein content was determined according to the Lowry method.

Western blot for the B2R was performed as previously described [46]. Equal amounts of protein (100 μg/lane) were separated using 10% SDS-PAGE under reducing conditions and transferred to nitrocellulose membranes (Biorad, Hercules, CA), blocked with 5% nonfat dry milk in PBS-0.1% Tween-20 buffer (PBS-T) and incubated overnight at 4°C with the same primary antibody used in immunohistochemistry: anti-B2R mouse monoclonal (1:1000, BD Transduction Laboratories, USA) diluted in blocking buffer. The membranes were washed six times for five minutes in PBS-T buffer, incubated with HRP-conjugated anti-mouse or anti-rabbit secondary antibodies (both 1:3000, Biorad, Hercules, CA) for one hour at room temperature and developed with chemiluminescence reagent (NEL-103, Western Lighting, Perkin-Elmer, MA) [30]. Membranes were exposed to CLxPosure film (Pierce, Rockford, IL). Equal protein loading was confirmed with Ponceau-S red staining (Sigma, St. Louis, MO). Images were scanned at 16-bit/600 dpi resolution with an Epson Perfection 3490 scanner (Epson Corporation, CA), saved as tiff files and calibrated to an optical density scale. The integrated optical density of bands was quantitated using the ImageJ v.1.34 software. The optical densities were expressed as the ratio of treatment/control.

### Proliferation and viability assays

Cells were seeded at a density of 80,000 cells/well on cover slides in 24-well plates and incubated for 18 hours in medium 1% FBS with and without 10.0 μMol/L BK (Sigma-Aldrich). Immunocytochemistry was performed as previously described using antibody to Ki67, a nuclear protein expressed in the active phases of the cell cycle. The proliferation index was represented by the percentage of cells with positive staining within total cells; the proliferation ratio represented the proliferation index of the treated/untreated cells in each experiment.

In addition, cell viability was evaluated using the MTS assay (Promega). Cells were placed in complete medium at a concentration of 15,000 cells/well in 96-well plates for 24 hours. To determine the effect of BK 10.0 μmol/L at different periods (0, 6, 18, 24 hours) cells were incubated in reduced serum with 1% FBS; the release of formazan was read after 2 hours of incubation with the MTS substrate at 495 nm by spectrophotometry (Multiskan EX, LabSystem).

All experiments were performed in triplicate and repeated at least three times.

### Migration assay

Cell migration was studied using the wound-healing assay under the effect of bradykinin, the B1R agonist [Lys-des-Arg^9^] bradykinin (LDBK) and the B1R or/and B2R antagonists. HTR-8/SVneo cells (100,000) were seeded in each well on cover slides in 24-well plates containing 500 μl complete medium for 24 hours. Cells were then washed 3 times with PBS and the wound was generated by removing cells in the center of the well with a sterile pipette tip; the unattached cells were washed away with PBS. The experiments were performed in reduced serum (1% FBS). Initially incubations in the different experimental conditions were carried out for 6, 18 and 24 hours. We then incubated for 18 hours, time in which maximal differences among treatments were observed. Cell migration was studied under the effect of 10.0 μMol/L of bradykinin and of 10.0 μMol/L of the B1R agonist LDBK. In addition cells were preincubated for 30 minutes with B1R or/and B2R antagonists: 10.0 μMol/L of Lys-(des-Arg^9^-Leu^8^) bradykinin (AB1R) and 10.0 μmol/L of D-Arg-Arg-Pro-Hyp-Gly-Thi-Ser-D-Tic-Oic (HOE-140, Sigma-Aldrich), and then incubated with their respective ligand for 18 hours in all assays.

The migration assay was performed 4 times in duplicate. Three images were obtained along the wound with a Nikon TMS inverted microscope connected to a Nikon CoolPix 4500 camera and quantified using ImageJ v.1.34 software. The migration index was defined as cells migrating in response to each study condition divided by cells migrating in response to medium alone [43].

### Invasion assay

Briefly, transwell inserts containing membranes with 8 μm pore size (Nunc) were coated with matrigel (BD Biosciences) as per the manufacturer's instructions. Cells (30,000) were spread over the matrigel (1:10) in 200 μl of medium with reduced serum (1% FBS), and 800 μl of culture medium was added to the lower chamber. Cell invasion was studied under the effect of BK, of BK plus two B2R antagonists-HOE-140 and the non-peptide antagonist bradyzide (BDZ, Sigma)-and finally with the B1R agonist LDBK plus its antagonist AB1R; BK, agonists and antagonists were all used at 10.0 μMol/L. The cells were preincubated with the antagonists for 30 minutes and then with their respective ligands for 18 hours. Bradykinin, the B1R agonist, and the antagonists were added on the upper and lower chambers of the invasion well.

For assessing the number of invaded cells, the filters were stained with anti-cytokeratin antibody and haematoxylin and mounted on cover slides with Kaiser's, glycerin-gelatin (Merck). Each experiment was performed in duplicate and was repeated 4 times. The inserts were examined by a blinded observer with an AX.10 microscope (Carl Zeiss) attached to a Nikon CoolPix 4500 camera; 20 photographs, were obtained per insert, and the number of cells in the underside of the filter was counted. Results are expressed as invasion index, where the level of invasion was defined as cells invading in response to BK divided by cells invading in response to medium alone [43].

### Gelatin zymography

Gelatinase activity was detected by zymography using methods described previously, with some modifications [46]. Cells were seeded at a density of 100,000 on plates in complete medium and incubated until 70% confluency was achieved; cells were further incubated in medium with reduced serum (1% FBS) for 24 h. BK was then added for 18 h. Aliquots from conditioned medium were resolved under non-reducing conditions in a 10% polyacrylamide gel containing 1.0 mg/ml gelatin (porcine skin, 300-bloom, Sigma, St. Louis, MO). After electrophoresis, the gels were washed twice at room temperature for 30 minutes in 2.5% Triton X-100, subsequently washed in buffer containing 50 mM Tris-HCl, 150 mM NaCl, 5 mM CaCl_2_, 1 μM ZnCl_2_, 0.05% Brij-35, 0.02% NaN3 at pH 7.5 and incubated in this buffer at 37°C for 24 hours. Thereafter, the gels were stained with 0.5% (w/v) Coomassie brilliant blue R-250 (Sigma-Aldrich) for 30 minutes, lightly destained in methanol:acetic acid:water (3:1:6) and finally stored in 5% acetic acid. Identification of each gelatinase band was done in accordance to the molecular weight, using purified human recombinant pro-MMP-2 and pro-MMP-9 (Calbiochem, USA) as standard (0.5 ng). Gels were scanned in the transmissive mode at 16-bit color/600 dpi (Epson Perfection 3490, Epson, CA) and stored in tiff format. Images were processed extracting the blue channel signal, converted to black and white and inverted for quantitation of the integrated optical density of gelatinolytic activities using the ImageJ v.1.34 software.

### Fluorescence Studies

Immunofluorescence was performed for B2R. Cells were seeded at a concentration of 80,000 cells/well on cover slides in 24-well plates for 24 hours and washed 3 times with cold PBS and fixed with methanol at -20°C for 20 minutes. Then washed with buffer PBS and incubated with protein block (Cas-Block, Zymed Laboratories, USA) serum free for 30 minutes in a humidified chamber. Cells were incubated with anti-B2R (anti-B2R 1:1000) overnight. Cells were washed twice for 5 minutes with PBS/Tween 20 0.1% and PBS, and incubated for 2 hours with anti-mouse-Rhodamine (PBS/1%, BSA) (1:40) (Pierce, USA). The nuclei were stained with DAPI (Pierce, USA) and the sections were mounted with Vectashield (Vector Laboratories, Inc, USA). Microphotographs were obtained with an AX.10 microscope (Carl Zeiss) attached to a Nikon CoolPix 4500 camera adapted with an epi-ilumination mercury lamp (MBO 100, Leistungselektronik JENA GmbH).

The identification of morphological changes in the actin cytoskeleton was performed using FITC-conjugated phalloidin (Molecular Probes). Briefly, 10,000 cells were seeded on coverslips, and incubated with culture medium with BK (10.0 μMol/L), BK plus preincubation with HOE-140 (10.0 μMol/L) and control conditions for 18 hours. The cells were rinsed with PBS and fixed in 4% paraformaldehyde in PBS for 15 minutes at room temperature. To quench the excess of aldehyde, 0.1 M glycine in PBS was added for 5 minutes. Cells were permeabilized with 0.1% Triton-X100 in PBS for 1 min and incubated with FITC-labelled phalloidin (1:100) in PBS for 15 minutes, finally cells were rinsed 3 times for 5 minutes in PBS, and mounted for microscopy with Vectashield. As above, microphotographs were obtained with an AX.10 microscope attached to a Nikon CoolPix 4500 camera adapted with an epi-ilumination mercury lamp. A blinded observer counted the number of filopodia connecting two cells in one 115-μm^2 ^rectangle per coverslip in at least 10 images from each experiment. The total number was then determined with ImageJ software, version 134. In addition, images were complemented by confocal laser scanning microscopy with an Olympus FluoView 1000 (Olympus UK).

### Statistical Analysis

Results are expressed as mean ± SE. One-way analysis of variance, followed by Tukey's Multiple Comparison post-hoc test, was used to test for differences between the different interventions and study periods with SPSS v10 (SPSS Inc.). A *P *< 0.05 was considered significant.

## Results

### Immunocytochemical detection of kininogen, kallikrein, the B1 and B2 receptors in HTR-8/SVneo cells

**Figure 1:**
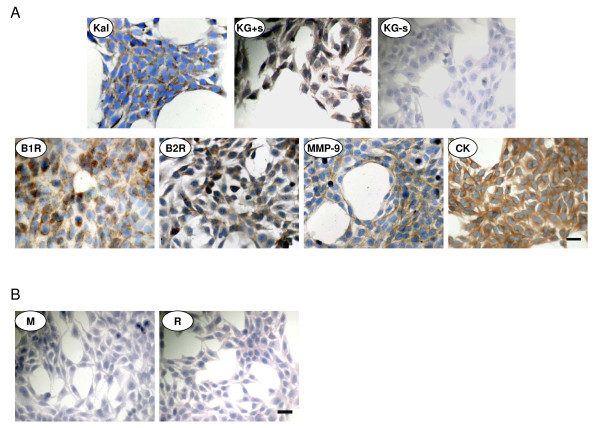
**Immunocytochemistry analysis**. A. Expression of Kal, kininogen (in cells incubated with [KG+s] or without serum [KG-s]), the B1R, B2R receptors, MMP-2 and MMP-9 in HTR-8/SVneo cells. B. Cells were cytokeratin (CK) positive. Negative controls with cells incubated with mouse (M) IgG serum (1:50) and rabbit (R) IgG fraction (1:50). Bar = 100 μm.

All cells expressed the components of the KKS (kallikrein, the B2R and the B1R), as well as MMP-2 and MMP-9. The staining pattern for Kal, the B2R and MMP-9 was diffuse, punctuate for B1R and granular for MMP-2. Cells incubated in the presence of serum expressed punctuate immunostaining for kininogen; however no signal was obtained when cells were deprived of serum for 24 hours. (Figure 1A). This finding supports the view that *in vivo *endogenous bradykinin likely derives from Kal acting on trapped circulating kininogen.

All cells were cytokeratin positive. Negative controls with cells incubated with mouse IgG serum and rabbit IgG fraction yielded no staining. (Figure 1B).

### Effects of bradykinin and its antagonists on the migratory capacity of HTR-8/SVneo cells

**Figure 2:**
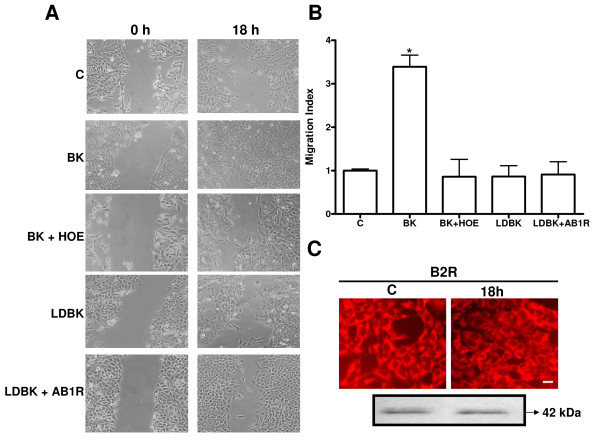
**Migration assay**. A. Representative images obtained along the wound, at 0 and 18 hours of stimulation with BK, the B1R agonist LDBK, and of preincubation with the antagonists of the B2 and the B1 receptors, HOE-140 and AB1R respectively (× 100). B. Effect of BK on the migratory capacity of HTR-8/SVneo cells analyzed at 18 hours. Mean ± SE; *p < 0.001 when comparing BK stimulation to control, and BK stimulation with preincubation with the B2R antagonist HOE-140; incubation with the B1R agonist LDBK, with or without preincubation with its antagonist AB1R did not modify cell migration. N = 4 for each condition. C. Representative immunofluorescence of the B2R, and of Western blot of lyzed cells, both after 18 hours of incubation under control conditions and BK stimulation.

The migration observed after incubating with BK (10.0 μMol/L) for 6 hours was minimal and showed no differences among the experimental conditions. At 24 hours wounds in all conditions were closed. However, observations at 18 hours showed that BK induced a 3-fold increase in the migration index as compared to controls (3.39 ± 0.27 versus 1.00 ± 0.04; *P *< 0.001; n = 4, respectively), and was completely blocked with the B2R antagonist HOE-140 (0.86 ± 0.39; *P *< 0.001; n = 4) On the other hand, incubation with the B1R agonist LDBK, with or without preincubation with its antagonist AB1R, had no effect on the migratory capacity of HTR-8/SVneo cells. (Figure 2A and 2B).

We considered that wound healing was due to cell migration, and not to proliferation, given that 10 μMol/L BK for 18 hours did not change the proliferation ratio between treated and untreated cells (1.06 ± 0.03, n = 3). No changes were observed in the content of the B2R at 18 hours, which yielded bands with a molecular weight of approximately 42 kDa (Figure 2C). The absence of differences in cell proliferation, viable cells and expression of the B2R between control conditions and BK stimulation supports that bradykinin stimulation enhances migration through its binding to the B2R.

### Effects of bradykinin and its antagonists on the invasive capacity of HTR-8/SVneo cells

**Figure 3:**
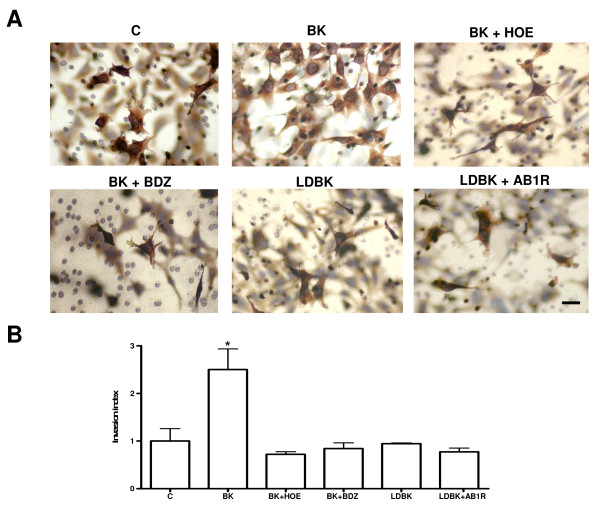
**Invasion assay**. A. Representative images of invaded inserts showing the effect of BK, the B1R agonist LDBK, and of preincubation with the antagonists of the B2 and B1 receptors, HOE-140/BDZ and AB1R respectively, on the invasive capacity at 18 hours, evaluated by the number of cells focussed on the underside of the filter. Blurry cells correspond to non-invading cells in the upper surface of the filter. B. Effect of BK and LDBK on the invasive capacity of HTR-8/SVneo cells analyzed at 18 hours, with and without preincubation with the B2R antagonists (HOE-140/BDZ] and the AB1R. N = 4 for each condition. Mean ± SE; *p < 0.05 vs. control and HOE-140. Bar = 100 μm.

Bradykinin (10 μMol/L) in the culture media of the upper and the lower chambers of the invasion well increased the invasion index by 2-fold at 18 hours as compared to controls (2.50 ± 0.44 vs.1.00 ± 0.3; *P *< 0.05; n = 6). This increase was totally abolished by preincubation with B2R antagonist HOE-140 (0.72 ± 0.05; *P *< 0.05; n = 3). Incubation with the B1R agonist LDBK, in the absence or presence of its antagonist AB1R for 30 minutes, had no effect on the invasive capacity of the cells (Figure 3A and 3B).

### Effect of bradykinin on the formation of filopodia in HTR-8/SVneo cells

**Figure 4:**
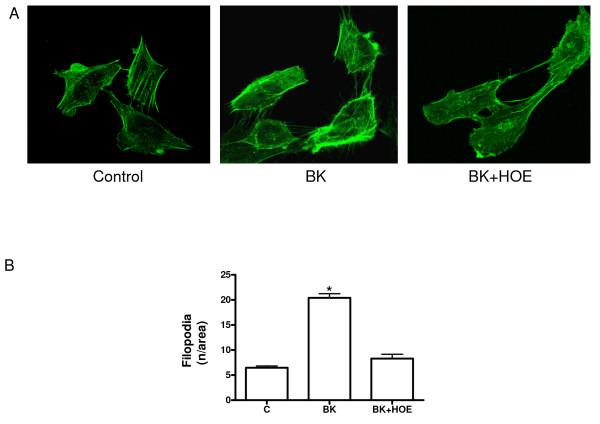
**Formation of filopodia in HTR-8/SVneo cells**. A. Representative images of filopodias in the area connecting two cells after 18 hours of stimulation with BK (10.0 μMol/L), and with BK (10.0 μMol/L) plus the antagonist of the B2R, HOE-140 (10.0 μMol/L) obtained with a laser scanning confocal microscope. B. Effect of BK and BK plus HOE-140 on the number of filopodia. Mean ± SE; *p < 0.001 vs. control and HOE-140, n = 3. Bar = 100 μm.

In areas connecting two cells, BK (10.0 μMol/L) induced a 3-fold increase in the number of filopodia at 18 hours as compared to controls (20.4 ± 0.8 vs. 6.47 ± 0.4; *P *< 0.001; n = 3). This effect was abolished when the cells were preincubated with the B2R antagonist HOE-140 (8.3 ± 0.8; *P *< 0.001, n = 3) (Figure 4A and 4B). This finding confirms the effect of the B2R on the modifications of the actin cytoskeleton that contribute to migration.

### Effect of bradykinin on the release of MMP-2 and MMP-9

**Figure 5:**
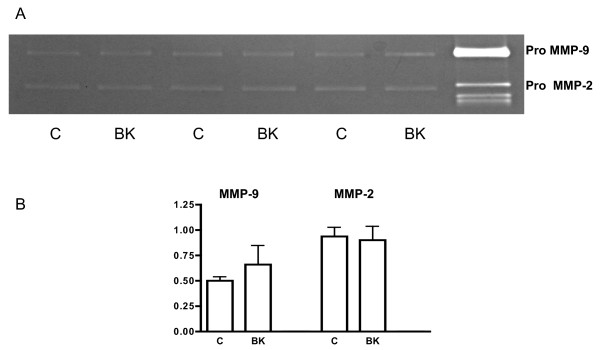
**Gelatinase activity of the culture media of HTR-8/Svneo cells**. A. Representative zymogram from conditioned media of HTR-8/SVneo cells cultured for 18 hours under control conditions (C) and with BK (10.0 μMol/L) from three different experiments; all lanes were loaded with equivalent amounts of proteins (25 μg). Human recombinant pro-MMP-9 and pro-MMP-2 were used as standards. B. Densitometric analysis. Mean ± SE; n = 3, N.S.

The gelatinase activity of MMP-9 and MMP-2 in the culture media showed no variation when incubated with BK for different periods (2 minutes, 2, 4, 6, 18, 24 and 48 hours), or concentrations (1.0, 10.0 and 100 μMol/L). Figure 5 depicts the gelatinase activity of 3 experiments after 18 hours of incubation in control conditions (C) or under stimulation with BK 10 μMol/L.

## Discussion

To our knowledge, this is the first report demonstrating an action of bradykinin on trophoblast migration and invasion, effects which can be attributed solely to activation of the B2R. In addition, this study adds kallikrein and the B2 receptor to the many factors shared by the human trophoblast and HTR-8/SVneo cells, underscoring their similarities. Though the promigratory and proinvasive effects of B2R stimulation were observed in immortalized HTR-8/SVneo trophoblasts, which are not genetically identical to primary CTB or EVT [47], and further studies are needed in human trophoblasts, the expression of the B2R in human extravillous trophoblasts [16] supports its participation in the invasive phenotype of genuine trophoblasts.

In order to elucidate the effect of bradykinin on cell migration/invasion, it is necessary to consider migration and invasion in the context of the repetitive conformational changes of the cytoskeleton involved in these processes [48]. The initial migratory response of a cell is to polarize and form cellular protrusions, such as actin microspikes, filopodia and lamellipodia, which push forward the leading edge of the cell to invade the surrounding tissue. The formation of these protrusions requires the polymerization of actin, and their stability depends on adherence to the extracellular matrix and the adjacent cells via transmembrane receptors linked to the actin cytoskeleton [27,49]. The increase in the number of filopodia in HTR-8/SVneo cells incubated with bradykinin confirmed these modifications of the actin cytoskeleton.

It has been shown that the formation of filopodia, actin microspikes, and lamellipodia are dependent on Cdc42 and Rac1. In concordance with our observations in HTR-8/SVneo cells, bradykinin promotes the formation of Cdc42-dependent membrane protrusions in fibroblasts [33], as well as peripheral actin microspikes and membrane ruffles with a temporal pattern similar to that observed with Cdc42Hs [48]. In human umbilical vein endothelial cells, Cdc42 is specifically activated by bradykinin [50]. In endothelial progenitor cells, BK stimulates the formation of filopodia and recruitment of Rac1 to the cell membrane; while the BK-induced polarization, formation of filopodia, and migration were inhibited by the B2R antagonist HOE-140 [29]. Part of the effects we observed after bradykinin stimulation could be mediated by the pro-invasive Cdc42 and Rac1-dependent responses provoked by PGE_2_, also present in HTR-8/SVneo cells [10,51,52], since the B2R mediates COX-2 induced PGE_2 _release in vascular smooth muscle and tumoral cells [53,54].

Apart from its conformational changes, a migrating cell must detach from the ECM and neighboring cells. In this regard, matrix metalloproteinases (MMPs) are believed to be a dominant system in trophoblast invasion [9]. Stimulation of the B2R induces MMP overexpression and cell migration in a rat astroglial cell line [39], while the B1R induces release of MMP-2 and MMP-9 in breast cancer cells [37]. Co-localization of MMP-9 and the B2R was observed in our previous studies in guinea-pig extravillous trophoblast [46]. The co-localization of MMP-9 and MMP-2 with the B2R was confirmed in HTR-8/SVneo cells by immunohistochemistry and enzymatic activity in this study. In spite of the functional and spatial associations between bradykinin and MMPs, no changes were observed in MMP-2 and MMP-9 activity in culture media after bradykinin stimulation.

The participation of plasminogen activators (PA) in BK enhanced invasion deserves further exploration. The B2R stimulates tissue (t)PA release from human endothelium [55], HTR-8/SVneo cells express urokinase (u)PA and tPA mRNA [36,40,41,56], uPA has been detected in extravillous trophoblasts, and gonadotrophin releasing hormones I and II, key facilitators of trophoblast invasion, are capable of up-regulating uPA [57].

Can the promigratory and invasive effect of BK be linked to what is already known about the modulation of the KKS system in pregnancy? In normal pregnant women the surge in urinary kallikrein reaches its maximum between 8 to 12 weeks, a stage of active trophoblast invasion [58]. On the contrary, reduced urinary kallikrein levels are observed in hypertensive pregnancies [59], and at 16 weeks represent one of the best predictors of preeclampsia [60]. Having demonstrated kallikrein and the B2R in cells involved in placentation in humans, guinea-pigs and rats [16,19-21,23,45,46,61,62], we hypothesize that a defective response of the KKS at the primordial target, the utero-placental interface could impair trophoblast invasion. This impairment could derive in part, from dimers of the angiotensin II type 1 and the B2 receptors observed in preeclampsia, which enhance the effect of angiotensin II and blunt the response to B2R stimulation [63,64]. Unfortunately, circulating levels of bradykinin are difficult to determine due to rapid degradation and artifactual generation of the peptide during sampling. Though these difficulties have been circumvented by determining a stable plasma metabolite-BK1-5-by liquid chromatography-tandem mass spectrometry [55], even if circulating levels could be titrated in normotensive and hypertensive pregnancies, the tissue bradykinin content would be impossible to define.

### Perspectives

This first report demonstrating a B2R-dependent stimulatory effect of bradykinin on trophoblast migration and invasion in an immortalized first trimester cell line supports the participation of the KKS in the local adaptations of pregnancy in a relatively recently described non-vasoactive role. Since the gene expression profile of HTR-8/SVneo is related-but dissimilar to primary CTB or EVT [47]-further studies, ideally in animals that share an analogous pattern of trophoblast invasion with humans, or *in vivo *complex models, as cocultured trophoblasts and endothelial cells or explants from early pregnancies with cognate outcomes are warranted [65,66].

## Competing interests

The authors declare that they have no competing interests.

## Authors' contributions

RE participated in the design of the study, performed the migration and the invasion assays, the statistical analysis and drafted the manuscript. JC performed the immunohistochemistry, and with FL did the Western blots. JC and GV conceived the study, participated in its design and coordination, and wrote the manuscript. All authors read and approved the final manuscript.
